# Inguinal Lymph Node Metastasis from a Sigmoid Stomal Cancer: A Case Report

**DOI:** 10.70352/scrj.cr.26-0363

**Published:** 2026-09-10

**Authors:** Kenji Shirakawa, Hironori Kobayashi, Takahiro Ishii, Kosuke Shibata, Norimasa Kuraoka, Kazuhiro Toyota, Raita Yano, Yasushi Hashimoto, Yoshihiro Sakashita, Yoshiaki Murakami, Katsunari Miyamoto

**Affiliations:** Department of Surgery, Hiroshima Memorial Hospital, Hiroshima, Hiroshima, Japan

**Keywords:** stomal cancer, inguinal lymph node metastasis, inguinal lymph node dissection, lymphatic drainage

## Abstract

**INTRODUCTION:**

Inguinal lymph node metastasis from colon adenocarcinoma is extremely rare. Cancer arising from a stoma is relatively rare, and cases with lymph node metastasis including inguinal lymph node metastasis are even rarer. Due to its rarity, there is no established surgical procedure for stomal cancer. Herein, we report a rare case of inguinal lymph node metastasis from a sigmoid stomal cancer, successfully treated by inguinal lymph node dissection.

**CASE PRESENTATION:**

An 87-year-old man had undergone abdominoperineal resection for rectal cancer when he was aged 28 years. He was referred to our hospital due to complaints of swelling and bleeding from the colostomy. Colonoscopy revealed a hemorrhagic type 2 tumor at the colostomy site. Biopsy revealed a moderately differentiated tubular adenocarcinoma. The diagnosis was adenocarcinoma occurring from a sigmoid colostomy. We performed a laparoscopic sigmoid colectomy with D3 lymph node dissection and colostomy reconstruction. The pathological stage was pT2N0M0 (pStage I). Contrast-enhanced CT performed 6 months postoperatively demonstrated enlargement of the previously noted left inguinal lymph node. Left inguinal lymph node dissection was performed because inguinal lymph node metastasis from sigmoid stomal cancer was suspected. Histopathological examination revealed moderately differentiated tubular adenocarcinoma. Because the histological features resembled those of the primary stomal cancer, the lesion was diagnosed as inguinal lymph node metastasis from sigmoid stomal cancer. Twenty-two months after the surgery, he has survived without recurrence.

**CONCLUSIONS:**

This case represents the first reported case of inguinal lymph node metastasis from a sigmoid stomal cancer without abdominal wall or skin invasion. Altered lymphatic drainage associated with long-standing stoma construction may have contributed to the development of inguinal lymph node metastasis, which might be treated by inguinal lymph node dissection.

## Abbreviations

CA19-9
: carbohydrate antigen 19-9
CEA
: carcinoembryonic antigen
IMA
: inferior mesenteric artery
LCA
: left colic artery
pT
: pathological primary tumor
UICC
: Union for International Cancer Control

## INTRODUCTION

Inguinal lymph node metastasis from colon cancer is extremely rare.^1,2)^ Cancer arising from a stoma is relatively rare, and cases with lymph node metastasis including inguinal lymph node metastasis are even rarer.^3–6)^ Due to its rarity, there is no established surgical procedure for stomal cancer, and the extent of lymph node dissection has not been determined. Herein, we report a case of inguinal lymph node metastasis from a sigmoid stomal cancer successfully treated by inguinal lymph node dissection and discuss its possible metastatic pathway.

## CASE PRESENTATION

**Fig. 1:**
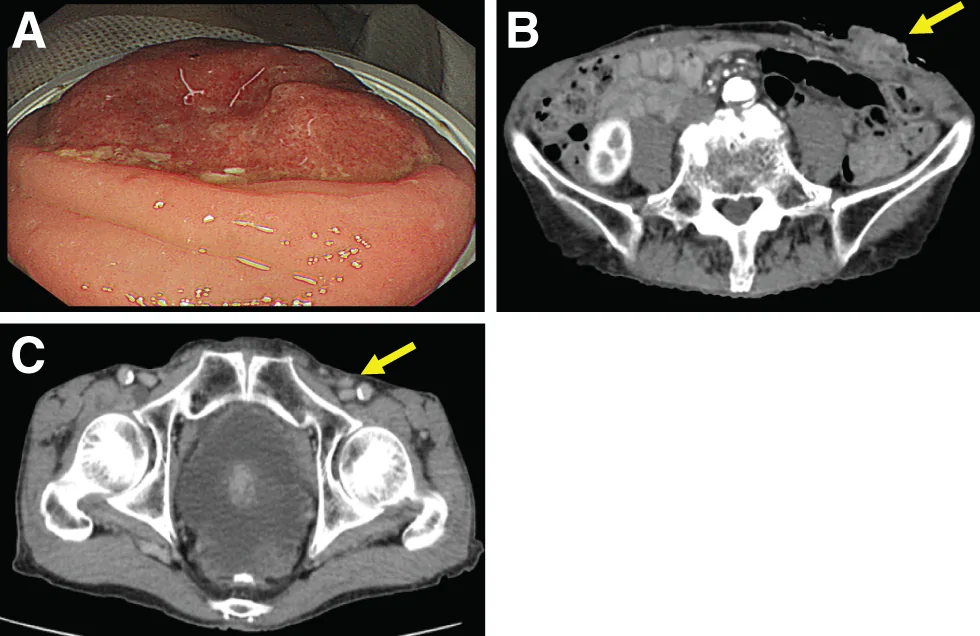
Colonoscopy and contrast-enhanced CT images. (**A**) Colonoscopy revealed a type 2 tumor at the colostomy site. (**B**, **C**) Contrast-enhanced CT revealed hypervascular irregular wall thickening at the stomal site (yellow arrow in **B**) and a moderately enlarged lymph node in the left groin (yellow arrow in **C**).

An 87-year-old man had undergone abdominoperineal resection for rectal cancer when he was aged 28 years. He was referred to our hospital due to complaints of swelling and bleeding from the colostomy. Colonoscopy revealed a hemorrhagic type 2 tumor at the colostomy site (**Fig. 1A**). Biopsy revealed a moderately differentiated tubular adenocarcinoma. The tumor marker levels were as follows: CEA, 8.8 ng/mL (upper reference limit, 5.0 ng/mL); CA 19-9, 10.6 U/mL (upper reference limit, 37.0 U/mL). Contrast-enhanced CT revealed hypervascular irregular wall thickening at the stomal site (**Fig. 1B**) and a moderately enlarged lymph node in the left groin, with no evidence of distant metastasis (**Fig. 1C**). The diagnosis was adenocarcinoma occurring from a sigmoid colostomy, and the clinical stage was cT3N0M0, cStage IIa (UICC, 9th edition).

**Fig. 2:**
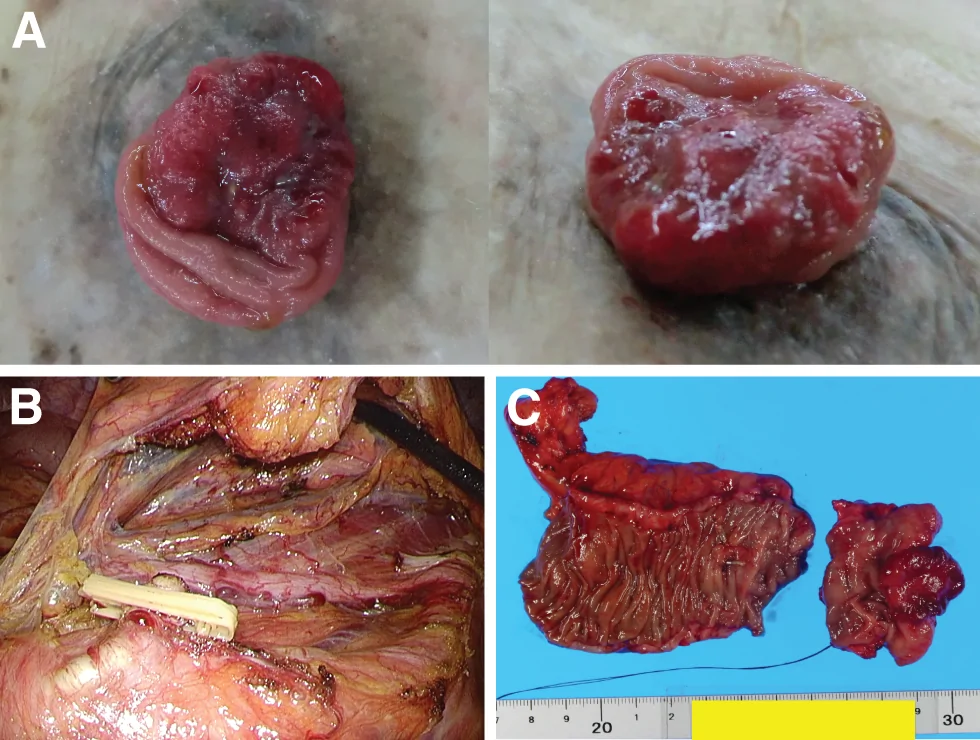
Surgical findings. (**A**) No tumor invasion into the skin was observed. (**B**) The IMA was dissected at its root, and a laparoscopic sigmoid colectomy with D3 lymph node dissection was performed. (**C**) Macroscopic findings of the resected specimen showed a 25 × 25-mm type 2 tumor in the sigmoid colostomy, with a proximal margin of 120 mm and a distal margin of 8 mm. IMA, inferior mesenteric artery

Based on the above preoperative diagnosis, we planned to perform a laparoscopic sigmoid colectomy with D3 lymph node dissection and colostomy reconstruction. Intraoperatively, the original colostomy was found to have been created via the transperitoneal route. First, the adhesion between the colostomy and the surrounding skin was dissected. No skin invasion was observed (**Fig. 2A**). Next, the colostomy was transected using a 45-mm purple Endo GIA Tri-Staple cartridge (Covidien, North Haven, CT, USA), and laparoscopic surgery was initiated. The IMA was divided at its origin, and laparoscopic sigmoid colectomy with D3 lymph node dissection was performed (**Fig. 2B**). Finally, the colostomy was reconstructed through the extraperitoneal route, and the surgery was completed. The operative time was 149 minutes, and the blood loss was 5 mL.

**Fig. 3:**
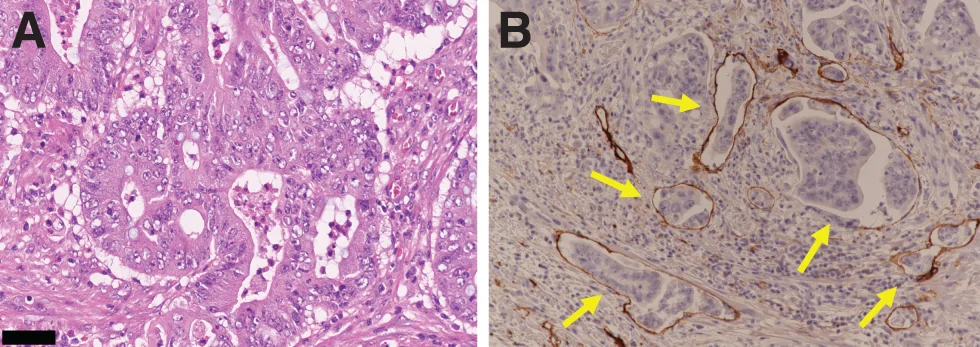
Histopathological and immunohistochemical findings. (**A**) The tumor primarily consisted of moderately differentiated tubular adenocarcinoma and poorly differentiated adenocarcinoma. (**B**) D2-40-positive lymphatic vessels with poorly differentiated adenocarcinoma embolization (yellow arrows) (× 240).

Histologically, the tumor was classified as adenocarcinoma, type 2, 25 × 25 mm, poorly differentiated non-solid > moderately differentiated tubular > papillary, pT2, intermediate infiltrative growth pattern, lymphatic invasion 1c, venous invasion 1a, no perineural invasion, negative pathologic proximal margin (120 mm), negative pathologic distal margin (8 mm), pathological regional lymph nodes 0, clinical distant metastasis 0, pStage I, curability A (UICC, 9th edition) (**Fig. 2C**). The tumor primarily consisted of moderately differentiated tubular adenocarcinoma and poorly differentiated adenocarcinoma (**Fig. 3A**). Numerous lymphatic vessel invasions were observed, and poorly differentiated adenocarcinoma embolization were identified (**Fig. 3B**).

**Fig. 4:**
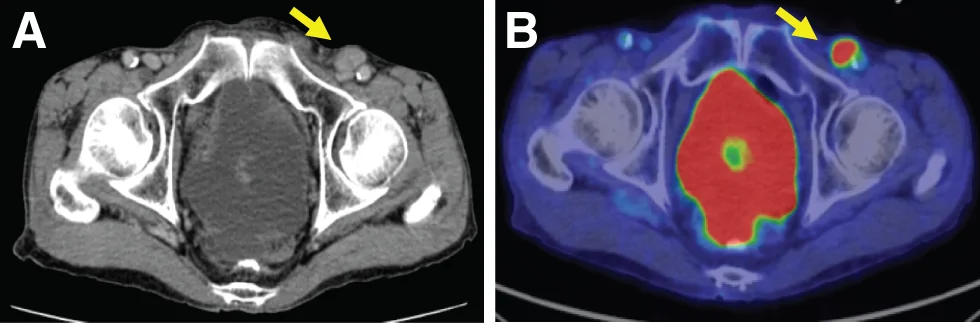
Findings of contrast-enhanced CT and PET/CT. (**A**) Contrast-enhanced CT revealed an enlarged lymph node in the left inguinal region (yellow arrow). (**B**) PET/CT scan revealing fluorodeoxyglucose uptake in the left inguinal lymph node only (yellow arrow).

Blood tests performed 3 months postoperatively showed that the CEA level had normalized. Contrast-enhanced CT performed 6 months postoperatively revealed enlargement of the previously noted left inguinal lymph node (**Fig. 4A**). PET/CT demonstrated intense fluorodeoxyglucose uptake at the same site, with a maximum standardized uptake value of 10.3, while no abnormal uptake was detected in other regions (**Fig. 4B**). CEA levels were elevated at 13.7 ng/mL, while the CA19-9 level was within the normal range at 10.2 U/mL. Based on the imaging findings and the elevated CEA level, left inguinal lymph node metastasis from the sigmoid stomal cancer was suspected.

**Fig. 5:**
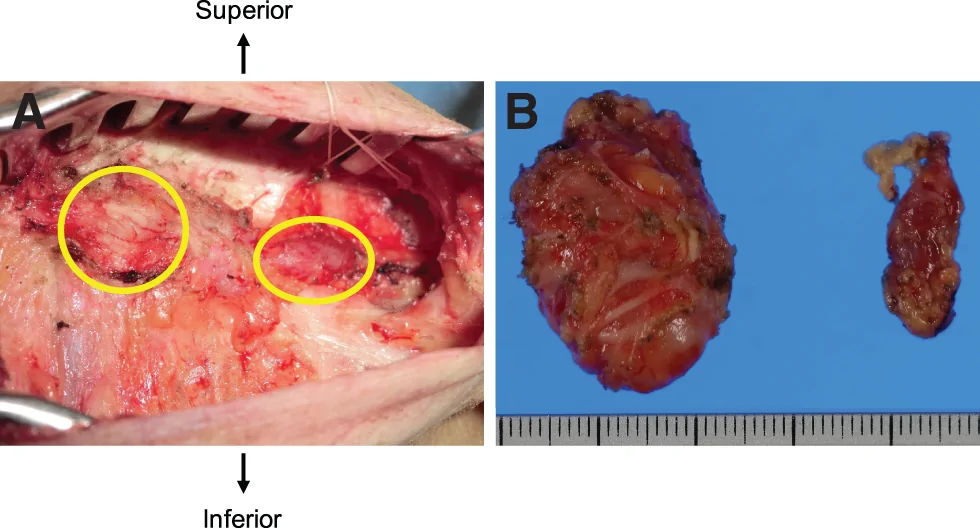
Surgical findings. (**A**) Two enlarged lymph nodes were noted in the left inguinal region (yellow circles). (**B**) The excised lymph nodes measured 3.0 × 2.5 cm (left side) and 2.0 × 0.8 cm (right side).

We performed a left inguinal lymph node dissection because the lesion was solitary. Two enlarged lymph nodes were identified (**Fig. 5A**). The operative time was 81 minutes, and the blood loss was 2 mL.

**Fig. 6:**
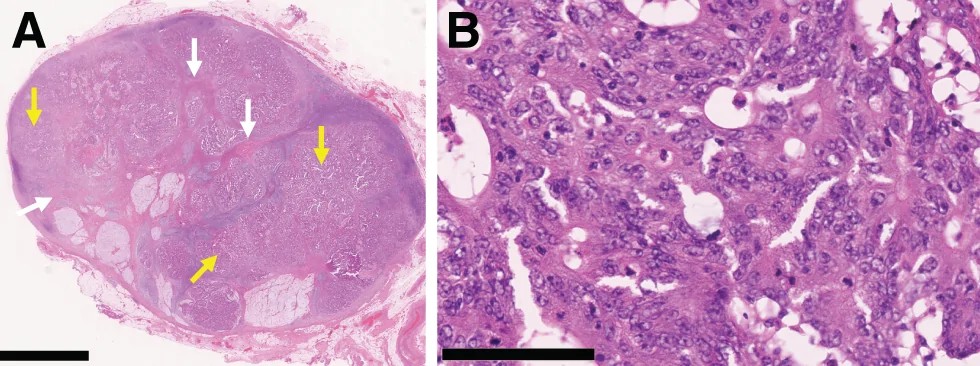
Histopathological findings. (**A**) The excised lymph nodes showed fibrosis (white arrows) and increased cancer cells (yellow arrows). The lymphoid architecture was destroyed (bar indicates 5 mm). (**B**) The cancer consisted of moderately differentiated tubular adenocarcinoma, which resembled the pathological appearance of the sigmoid stomal cancer (bar indicates 100 µm).

The excised lymph nodes measured 3.0 × 2.5 cm and 2.0 × 0.8 cm (**Fig. 5B**). Histopathological examination revealed moderately differentiated tubular adenocarcinoma in the former specimen (**Fig. 6A** and **6B**). The histological features resembled those of the sigmoid stomal cancer, so we diagnosed it as inguinal lymph node metastasis from stomal cancer. Blood tests performed 3 months after the second surgery showed normalization of the CEA level. Postoperative adjuvant chemotherapy was not administered at the patient’s request. Twenty-two months after the surgery, he has survived without recurrence.

## DISCUSSION

It is known that both early and late complications arise after ileostomy or colostomy procedures.^7)^ Although metachronous sigmoid colon cancer following rectal cancer is not uncommon,^8,9)^ carcinoma arising at a colostomy site is a rare late complication after stoma construction, and only a limited number of cases have been reported.^3)^ In previous reports, an adenoma–carcinoma sequence,^10)^ de novo development,^11)^ development of micrometastasis left behind in lymph nodes along the IMA pedicle,^12)^ cancer family syndrome,^13)^ and suspected implantation^14)^ have been reported as mechanisms for the development of stomal cancer. It is thought that multiple factors overlap in the development of stomal cancer, but the exact cause has not yet been identified. One reason why stomal cancer is rare is that the colostomy is not exposed for long periods to intestinal bacteria or feces, which are considered carcinogenic factors.^15,16)^

When we searched past reports, our case was the 9th reported case of lymph node metastasis from stomal cancer and the 4th reported case of inguinal lymph node metastasis from stomal cancer. Furthermore, our case was the first reported case of inguinal lymph node metastasis from stomal cancer without abdominal wall or skin invasion. Previous cases of lymph node metastasis from stomal cancer are listed in **Table 1**.^4–6,14,17–20)^ All cases of inguinal lymph node metastasis from stomal cancer including our case showed solitary lymph node metastasis. Lymph node dissection was performed in all cases of inguinal lymph node metastasis. Postoperative adjuvant chemotherapy was administered in 4 of the 9 cases; however, none of the 4 cases with inguinal lymph node metastasis received adjuvant chemotherapy. Lymph node metastasis is thought to be rare because radical surgery with lymph node dissection at the initial operation may alter lymphatic drainage, thereby making subsequent lymph node metastasis less likely.^6)^ Inguinal lymph node metastasis was observed in 4 cases including our case among 9 cases of stomal cancer with lymph node metastasis.^4–6)^ These findings indicate that, although lymph node metastasis from stomal cancer is rare, inguinal lymph node metastasis represents an important metastatic pattern in stomal cancer.

**Table 1:** Review of reported cases of stomal cancer with lymph node metastases

Author (Year)	Age/Sex	Past history (Age)	Original surgery	Differentiation	pT	LN metastasis	Treatment	Adjuvant chemotherapy	Outcome
Chintamani et al.^14)^ (2007)	30/M	RC (24)	APR	por 2	T4b (abdominal wall)	Paraintestinal LN	Bowel resection	Yes	DOD, 50 mo
Igari et al.^17)^ (2011)	80/M	RC (50)	APR	tub 2	T4b (abdominal wall)	Paraintestinal LN	Bowel resection	No	NED, 2 mo
Nabeyama et al.^4)^ (2013)	86/W	RC (58)	APR	tub 2	T4b (abdominal wall)	Inguinal LN	Inguinal LN dissection	No	NED, 6 mo
Iwamoto et al.^5)^ (2015)	82/W	RC (55)	APR	tub 2	T4b (skin)	Paraintestinal and inguinal LNs	Bowel resection and inguinal LN dissection	No	NED, 60 mo
Miyake et al.^6)^ (2015)	75/M	RC (50)	APR	tub 1, tub 2	T4b (abdominal wall)	Inguinal LN	Inguinal LN dissection	No	NED, 18 mo (lung met resected at 12 mo)
Yabe et al.^18)^ (2017)	60/M	FAP (30)	Subtotal colectomy and colostomy	tub 2	T2	Paraintestinal LN	Bowel resection	Yes	NED, 24 mo
McEnteee et al.^19)^ (2020)	65/W	RC (59)	APR	tub 2	T2	Paraintestinal LN	Bowel resection	Yes	NED, 18 mo
Choi et al.^20)^ (2023)	53/W	RC (47)	APR	NR	T2	Paraintestinal LN	Bowel resection	Yes	NED, 24 mo
Our case	87/M	RC (28)	APR	por 2, tub 2, pap	T2	Inguinal LN	Inguinal LN dissection	No	NED, 22 mo

APR, abdominoperineal resection; DOD, died of disease; FAP, familial adenomatous polyposis; LN, lymph node; M, men; mo, months; NED, no evidence of disease; NR, not reported; pap, papillary adenocarcinoma; por 2, non-solid type poorly differentiated adenocarcinoma; pT, pathological primary tumor; RC, rectal cancer; tub 1, well-differentiated adenocarcinoma; tub 2, moderately differentiated adenocarcinoma; W, women

Because lymph node metastasis of colon cancer typically occurs via mesenteric vessels, inguinal lymph node metastasis from colon cancer is considered rare.^21)^ According to previous reports, inguinal lymph node metastasis from colon cancer is thought to occur when tumor invasion of the abdominal wall allows spread through the superficial lymphatic pathways along the inferior epigastric artery.^5,21,22)^ Our literature search identified 3 reported cases of inguinal lymph node metastasis from sigmoid colon cancer without a stoma (**Table 2**).^1,21,23)^ Two cases were classified as pT4,^1,21)^ whereas the remaining case was pT3.^23)^ The pT3 sigmoid colon cancer case developed metachronous inguinal lymph node metastasis and was accompanied by synchronous abdominal wall metastasis at a previous cesarean section incision. It was hypothesized that tumor cells spread to the inguinal lymph nodes via the abdominal wall metastasis. In addition, a case of pT3 ascending colon cancer with inguinal lymph node metastasis has also been reported.^22)^ In that case, occult abdominal wall invasion or the development of an aberrant lymphatic pathway from the right colon was proposed as a possible explanation for the inguinal lymph node metastasis, although the exact mechanism remained unclear. Taken together, these reports suggest that inguinal lymph node metastasis from pT1–pT3 sigmoid colon cancer without a stoma is extremely rare.

**Table 2:** Review of reported cases of inguinal lymph node metastasis from sigmoid colon cancer without a stoma

Author (Year)	Age/Sex	Differentiation	pT	Stage	Timing of inguinal LN metastasis	Treatment
Pisanu et al.^21)^ (2011)	62/M	NR	T4b (abdominal wall)	T4bN1M0	Metachronous	Surgical resection
Tanabe et al.^23)^ (2019)	42/W	tub 1	T3	T3N2bM1 (abdominal wall)	Metachronous	Surgical resection
Motamiez et al.^1)^ (2024)	35/W	sig	T4b (ileum, ovary)	T4bN2bM0	Metachronous	Surgical resection

LN, lymph node; M, men; NR, not reported; pT, pathological primary tumor; sig, signet-ring cell carcinoma; tub 1, well-differentiated adenocarcinoma; W, women

In previous reports of stomal cancer with inguinal lymph node metastasis, skin or abdominal wall invasion was observed.^4–6)^ However, unlike previous reports, no skin or abdominal wall invasion was observed in our case (**Fig. 2A**). Our case showed no metastasis to paraintestinal lymph nodes or direct skin invasion but demonstrated high-grade lymphatic invasion (**Fig. 3B**). As discussed above, inguinal lymph node metastasis from sigmoid colon cancer is extremely rare under normal lymphatic drainage.^21)^ However, lymphatic drainage from the abdominal wall below the umbilicus flows to the superficial inguinal lymph nodes. Although speculative, adhesions and tissue changes associated with a long-standing colostomy may have resulted in the formation of new lymphatic pathways between the stoma and the surrounding skin or subcutaneous tissues. In the present case, tumor cells may have spread to the inguinal lymph nodes through this newly formed lymphatic pathway. Notably, no direct invasion of the peristomal skin was identified in the present case. Taken together, these findings suggest that altered lymphatic drainage associated with a long-standing colostomy may have contributed to the unusual metastatic pattern observed in the present case. Accordingly, the possibility of inguinal lymph node metastasis should be considered even in stomal cancer without apparent skin invasion.

Inguinal lymph node metastasis is associated with a poor prognosis for rectal or anal canal adenocarcinoma.^24,25)^ Non-regional lymph node metastasis is considered systemic metastasis, and chemotherapy is the basic treatment. However, several reports have described patients with inguinal lymph node metastasis from colon cancer who remained recurrence-free following inguinal lymph node dissection.^5,21,22,26)^ These findings suggest that surgical resection of inguinal lymph node metastases may improve the prognosis of selected patients with colon cancer. In all 3 previously reported cases of stomal cancer with inguinal lymph node metastasis, inguinal lymph node dissection was performed because R0 resection was considered feasible. Although recurrence was observed in one of the 3 previously reported cases,^6)^ all patients were alive at the time of publication, and 1 patient remained alive for 60 months.^5)^ In our case, the patient has remained recurrence-free for 22 months without postoperative adjuvant chemotherapy. None of the 3 previously reported cases of stomal cancer with inguinal lymph node metastasis received postoperative adjuvant chemotherapy. These findings suggest that surgical resection of inguinal lymph node metastases in stomal cancer may improve the prognosis of selected patients, although further accumulation of cases is needed to establish the optimal treatment strategy.

## CONCLUSIONS

This case represents the first reported case of inguinal lymph node metastasis from sigmoid stomal cancer without abdominal wall or skin invasion. Altered lymphatic drainage associated with a long-standing stoma may have contributed to the development of inguinal lymph node metastasis. Although lymph node metastasis from stomal cancer is rare, inguinal lymph node metastasis should be recognized as a possible metastatic pattern, which might be treated by inguinal lymph node dissection when R0 resection is achievable.

## DECLARATIONS

### Availability of data and materials

All data generated or analyzed during this study are included in this published article.
